# Bochdalek Hernia as a Discrepant Intraoperative Diagnosis in a Paraesophageal Hernia Surgery: A Surgical Case Report

**DOI:** 10.1155/cris/9953339

**Published:** 2026-02-24

**Authors:** Walter Abila Akello, I. M. Karani, W. O. Sibuor, Amos Washika

**Affiliations:** ^1^ Department of Medical Physiology, Egerton University, Njoro, Kenya, egerton.ac.ke; ^2^ Department of Surgery, St. Theresa Mission Hospital, Kiiru-Meru, Kenya

**Keywords:** Bochdalek hernia, fundoplication, hiatus hernia, paraesophageal hernia

## Abstract

Bochdalek hernia (BH) is a rare paediatric condition, but late diagnoses occur in adults. It occurs following incomplete posterolateral development of the diaphragm. Adult BH is asymptomatic in most cases, but it has the potential of being life‐threatening. This case report is of a 35‐year‐old female patient who presented to us with a history of childhood abdominal discomfort leading to frequent hospitalisation. Presently, she came with complaints of increasing abdominal pain, postprandial vomiting, and a 31% weight loss over 3 months before the current admission to our facility. A preoperative diagnosis of a Type IV hiatus hernia with a possible volvulus was made with the assistance of radiology, which turned out to be left BH intraoperatively. Only the herniated stomach was laparoscopically reduced due to technical difficulties and pexied along the defect to seal it. The postoperative period was unremarkable. This case report highlights the need for maintaining a high index of suspicion and surgical preparedness for BH in patients with high‐grade hiatus hernia.

## 1. Introduction

Congenital diaphragmatic hernias (CDHs) are due to embryologic failure of the diaphragm to fuse and are associated with neonatal lung hypoplasia, respiratory distress and vomiting. CDH affects 2 in 10,000 newborns, where 90% of CDHs are Bochdalek hernias (BH), 3% Morgagni, and 2% present as diaphragm eventration due to poor lateral myocyte migration, while 1% are due to central tendon defects [1].. The mortality rate of CDH varies globally, with high‐income countries (HICs) recording a 20% death rate in contrast to 90% in low‐ to middle‐income countries (LMICs) [1]. Even though BH is primarily a paediatric diagnosis, a retrospective review of 13,138 adult computed tomography (CT) scans identified 22 incidental diagnoses of BH, of which 17 were women [2]. This points to the underdiagnosed burden of subclinical BH, which occasionally may be symptomatic with increased intra‐abdominal pressure [3]. To the best of our knowledge, this constitutes the first reported case of left‐sided BH in an adult female patient in Kenya.

## 2. Case Presentation

A 35‐year‐old female patient presented to our facility with complaints of epigastric pain and refractory vomiting for the last 6 months. The patient reported a history of childhood mild ‘abdominal discomfort’, a series of endoscopies and treatment for gastritis. She describes the intensity of the epigastric pain over the last 3 years to be gradually increasing with no known alleviating factors. She reported vomiting every ‘5 min’ postprandially, a weight loss rate of 31% in 3 months, as well as pain rated 6/10 that made her seek medical attention.

Physical examination revealed BP of 156/80 mmHg, pulse of 90 b/m, oral temperature of 36.6°C, respiratory rate of 18 b/m and SPO_2_ of 93. Abdominal examination revealed significant scaphoid and epigastric tenderness on light palpation. No masses or organomegaly noted. The rest of the examination was benign. The patient’s laboratory values were WBC 5,810/μL with normal differentials, platelets 250,000/μL, Hb 11.4 g/dL, random blood sugar 5.7 , urea 1.71 and creatinine 80 μmol/L.

**Figure 1:**
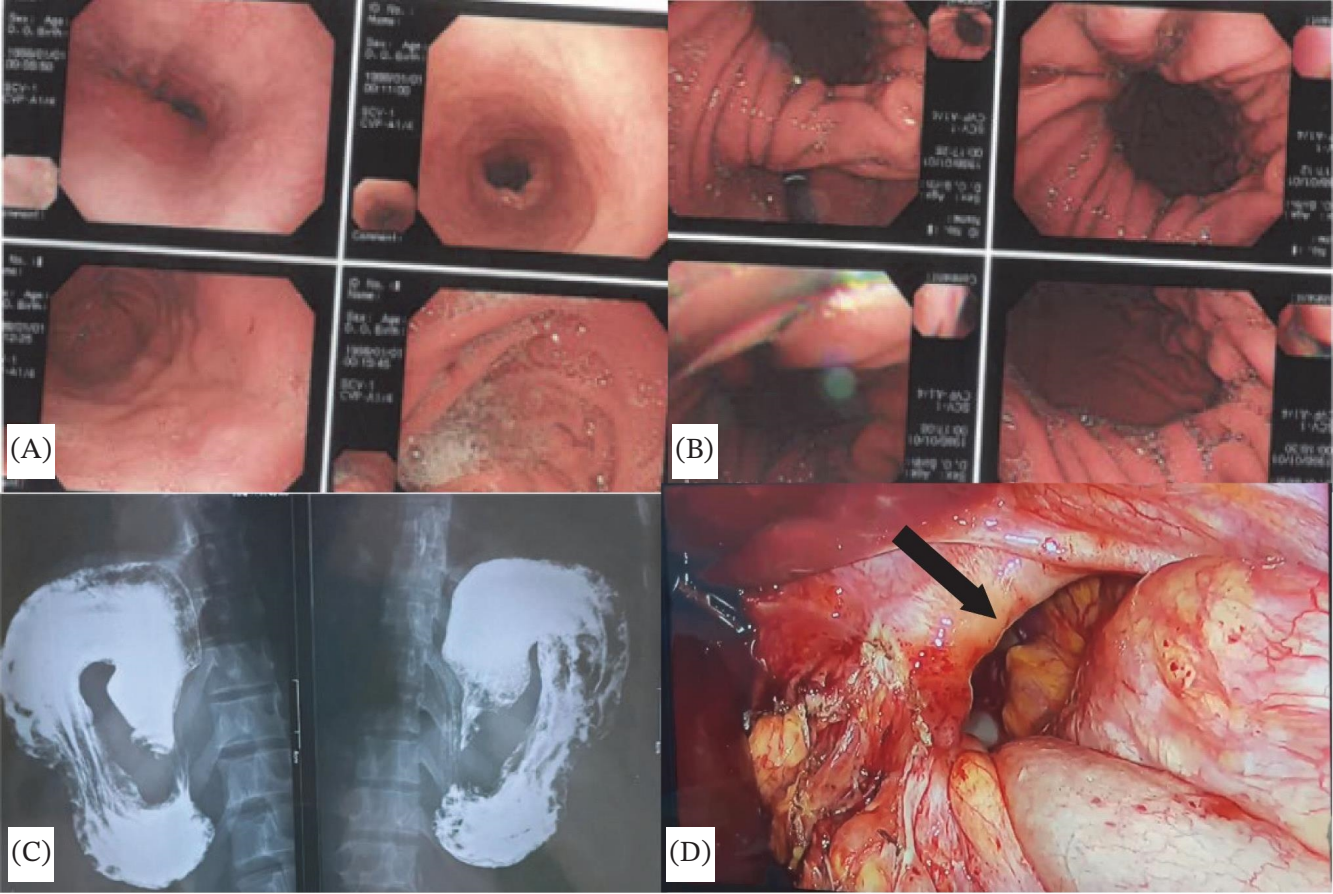
(A) Normal gastroesophageal junction at endoscopy. (B) Retroflexed view showing complete loss of the gastroesophageal fold and paraesophageal hernia. (C) Barium study showing inverted stomach. (D) Intraoperative finding showing the diaphragmatic defect (black arrow).

The upper gastrointestinal endoscopy performed in our facility revealed mid‐gastric constriction (Figure 1A), accompanied by herniation of the fundus and body of the stomach, visible on a retroflex view (Figure 1B). Access to the antrum and duodenum was challenging endoscopically. This prompted a barium swallow, which demonstrated a retroverted stomach in the lower chest, suggestive of organo‐axial gastric volvulus (Figure 1C). A preoperative diagnosis of a Type IV paraoesophageal hiatal hernia was made; however, a discrepant intraoperative diagnosis of left BH (Figure 1D) was made. The fundus and the body of the stomach, as well as the spleen, had herniated into the left chest cavity through a hemidiaphragmatic defect, but with a normal oesophageal hiatus. Due to technical difficulties in reducing the entire sac, only the stomach was mobilised and pexied along the defect to seal it.

**Figure 2:**
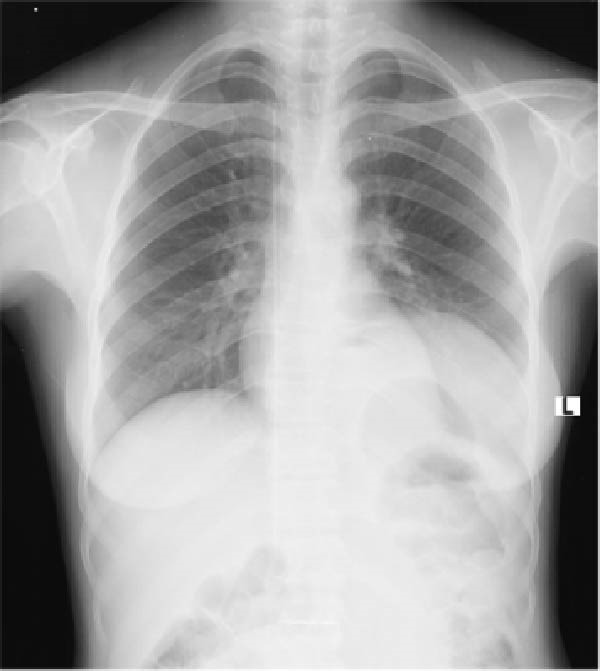
Postoperative X‐ray film shows an expanded left lung window, an improvement from the preoperative lung window.

The postoperative course was uneventful. Incentive spirometry was encouraged to prevent postoperative respiratory complications. Soft meals and early mobilisation were encouraged on day one postoperatively, and then they were discharged after 3 days in a stable condition. She was booked for the surgical outpatient clinic after 2 weeks. Fourteen days postoperative chest X‐ray showed increased left lung volume (Figure 2). She reported resolution of postprandial vomiting, and her weight had increased by 3.5 kg within 2 weeks. The patient is doing well as of date and continues on scheduled follow‐up.

## 3. Discussion

BH occurs when the prospective pleural and peritoneal cavity remains in open communication via the pericardioperitoneal canal (Bochdalek foramen) along the posterior walls. The closure of the left and right canals begins at the 5^th^ week of gestation through the extension of the pleuroperitoneal membrane towards the septum transversum and oesophageal mesentery, then fuses by the 7^th^ week of gestation. Failure of fusion of either the left or right periodioperitoneal canals results in BH. Left‐sided BH is 80% more prevalent because of the late closure of the left hemidiaphragm.

In this case, with the antral pylorus situated in the abdomen, the stomach’s normal capacity was compromised, thus increasing intragastric pressure. The mid‐gastric constriction likely disrupted gastric emptying. Consequently, high intragastric pressure, delayed emptying and vagal irritation possibly explain postprandial vomiting 5 min after eating. Complications can occur due to gastrothorax tension [4, 5], where gastric bloating causes intrathoracic pressure to rise, impeding venous return and interfering with the mechanics of breathing [6]. Therefore, diagnosis and surgical management of BH should be initiated promptly to prevent potential life‐threatening complications [7].

Diagnosing an adult congenital diaphragmatic hernia can be difficult because of its non‐specific symptoms, such as epigastric pain, vomiting, early satiety and respiratory distress. Clinical suspicion of BH in an adult should be considered when a patient shows both respiratory and gastrointestinal symptoms [8]. Chest X‐rays are typically used initially in diagnosis, revealing gas‐ or fluid‐filled intestinal loops, but their sensitivity is limited [7]. A CT scan confirms the diagnosis by showing soft tissues or fat above the diaphragm, a mass proximal to the diaphragm and continuous soft tissue density above and below the diaphragm [7]. However, imaging‐based diagnosis of BH remains challenging [9, 10]. In a systematic review of 173 studies involving BH, where most diagnostic methods were X‐ray and CT scan, 31.6% of all definitive diagnoses were made during surgery [7].

Surgery is the mainstay treatment of BH by reducing the hernia contents and closing the diaphragmatic defect [7]. The laparoscopic surgical approach is preferred over open surgeries in elective cases due to reduced hospitalisation stay [11, 12]. We adopted an abdominal laparoscopic approach to reduce the hernia content with a gastropexy around the defect as a stopgap measure due to technical difficulties, but this is not standard of care. The patient had a desirable postoperative outcome, that is, was discharged 3 days postoperatively, vomiting resolved, achieved a weight gain rate of 8.1% 14 days postoperatively and a repeat X‐ray indicated an increased left lung volume.

**Figure 3:**
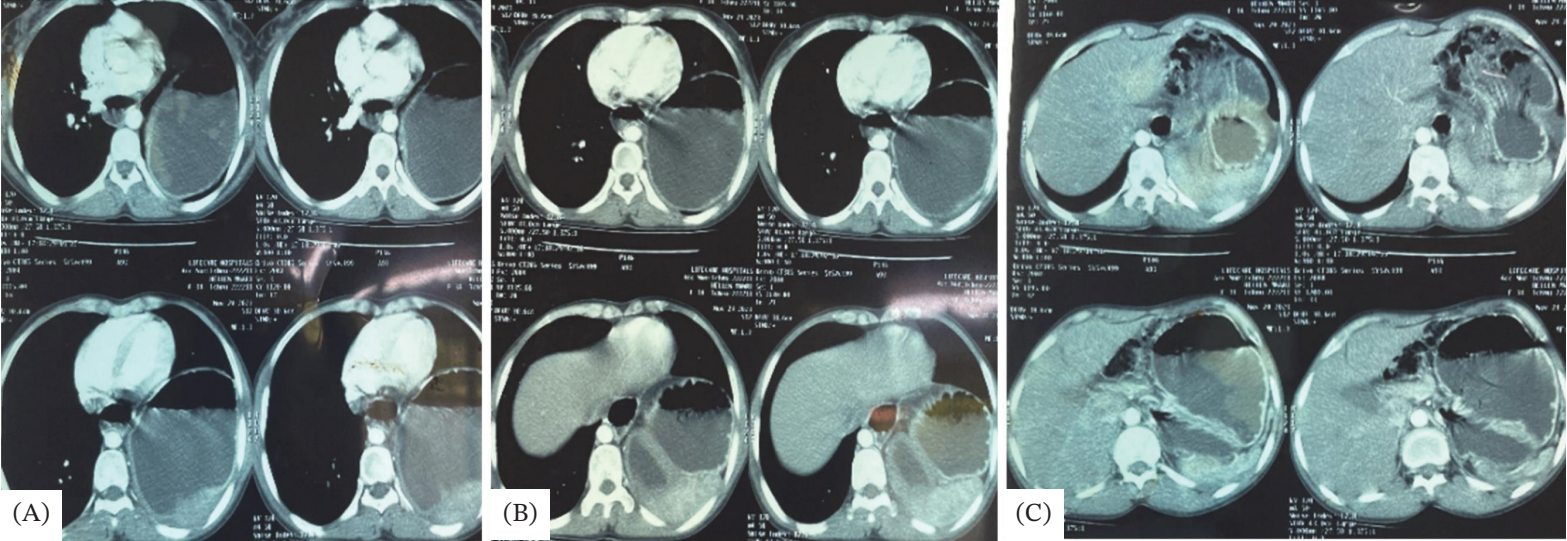
(A) Axial CT scan showing the stomach in the left chest cavity. (B) Shows diaphragmatic constriction. (C) Shows the distal body of the stomach and the antrum.

This case report had several limitations. First and foremost, repeat confirmatory preoperative CT was not done in the present admission of the patient. A CT scan done 8 months prior to the current admission was suggestive of diaphragmatic hernia (Figure 3). Second, the laparoscopic surgical approach used was meant for the correction of paraoesophageal Type IV hernia, but an incidental intraoperative diagnosis of BH was made involving part of the stomach and a non‐strangulated spleen. The surgical team prioritised gastric reduction without complete reduction of the spleen for a twofold reason: technical difficulties reducing the large hernia sac and repairing the defect. Gastric reduction was meant to prevent the risk of volvulus and gastrothorax tension and to allow feeding.

## 4. Conclusion

BH is rare in adults and often remains subclinical, but it can be life‐threatening. In this case, the diagnosis had been both delayed and missed entirely, and subsequently, treatment was delayed. This highlights the conundrum of BH diagnosis. The risk of gastrothorax tension among BH patients with a herniated stomach is high, which can lead to significant cardiorespiratory complications. Therefore, a high index of suspicion is necessary when gastrointestinal and respiratory symptoms co‐occur, especially when supported by suggestive CT scans. Surgery should be performed even in asymptomatic adult BH cases.

## Author Contributions

**Walter Abila Akello**: writing – original draft preparation, resources, writing – review and editing. **Amos Washika and I. M. Karani:** writing – review and editing. **W. O. Sibuor**: conceptualisation, writing – review and editing.

## Funding

No funding was received for this manuscript.

## Consent

Written informed consent was obtained from the patient for the publication of this case report and accompanying images.

## Conflicts of Interest

The authors declare no conflicts of interest.

## Data Availability

The data are available on request due to privacy/ethical restrictions.
